# Comparison between open reduction and internal fixation and minimally invasive surgery in management of Sanders type II calcaneal fracture: A randomized controlled trial protocol

**DOI:** 10.1097/MD.0000000000023813

**Published:** 2020-12-18

**Authors:** Dingshan Xue, Baozhen Lou, Rongrong Tan, Hongchang Yu

**Affiliations:** a Department of Orthopaedics; b Department of Anesthesiology, PLA Army 80th Group Military Hospital, Shandong, China.

**Keywords:** minimally invasive surgery, open reduction and internal fixation, prospective, protocol, Sanders type II calcaneus fracture

## Abstract

**Background:**

The minimally invasive surgery possesses an essential and growing function in treating the calcaneal fractures, but the related literature on this topic is limited. For our study, the main purpose was to compare the early prognosis of a group of the patients with Sanders type II fracture of calcaneus treated via minimally invasive surgery and open reduction and internal fixation (ORIF).

**Methods:**

This is a prospective randomized controlled trial in the patients who suffer from displaced intra-articular calcaneal fractures. This current study was carried out in accordance with the guidelines of “CONSORT statement” for the randomized controlled studies. All patients were randomly assigned into 2 groups on the basis of a random number table, namely the minimally invasive treatment group and the ORIF group using conventional methods. Inclusion criteria included the followings: aged between 18 to 59 years old; closed and unilateral fracture; patients with displaced intra-articular calcaneal fracture (>2 mm) involving Sanders Type IIC and Type IIB; and patients have enough mental capacity to understand and answer questions in the evaluation scale. In the process of outpatient follow-up, the radiographs were taken at 1, 3, 6, and 12 months. The functional results involved the American Orthopaedic Foot and Ankle Score, Foot Function Index, and the pain score.

**Conclusions:**

This protocol will give us research directions in future work.

**Trial registration:**

This study protocol was registered in Research Registry (researchregistry6261).

## 1 Introduction

Calcaneal fractures are the most prevalent tarsal fractures, which accounts for 60 percent of all the tarsal fractures, of which 75% are intra-articular fractures.^[1]^ Despite the management of calcaneal fractures is still controversial, it is reported that the nonsurgical treatment is less effective for the intra-articular calcaneal fractures with abnormal posterior foot morphology and subtalar arthritis. Hence, many authors consider surgical treatment rather than the conservative treatment for such fractures.^[2,3]^

Open reduction and internal fixation (ORIF) containing the conventional plate fixation through the expandable lateral approach with L-shape is regarded standard for management of the displaced intra-articular fractures of calcaneum, as it offers superior fracture exposure and direct compression of the rectavelled posterior facet fragments.^[4–6]^ However, some former studies have indicated a high incidence of wound complications after operation, involving the necrosis of wound edge, hematoma, debridement, and deep infection.^[7–10]^ Because of these risks, more and more surgeons have focused on protecting soft tissue over the past 20 years. Through the efforts of most orthopedic specialists, a number of less invasive techniques have been developed to replace ORIF, including percutaneous cannulated screws, percutaneous K lines, percutaneous plate positioning, and arthroscopy.^[11,12]^ Therefore, many current intra-articular calcaneal fractures can be treated with minimal soft tissue dissection rather than traditional ORIF and plate construction.

The minimally invasive surgery possesses an essential and growing function in treating the calcaneal fractures, but the related literature on this topic is limited. For our study, the main purpose was to compare the early prognosis of a group of the patients with Sanders type II fracture of calcaneus treated via minimally invasive surgery and ORIF.

## 2 Material and method

### 2.1 Study design

This is a prospective randomized controlled trial in the patients who suffer from displaced intra-articular calcaneal fractures. This current study was carried out in accordance with the guidelines of “CONSORT statement” for the randomized controlled studies. All patients were randomly assigned into two groups on the basis of a random number table, namely the minimally invasive treatment group and the ORIF group using conventional methods. These operations were implemented by a team of 2 experienced surgeons. All the radiologic evaluations were performed via 2 independent experienced radiologists. The study protocol was approved by the institutional review committee of the PLA Army 80th Group Military Hospital (No. 2019089). Prior to the registration began, our current research was registered in the Research Registry (researchregistry6261).

### 2.2 Patient enrollment

Inclusion criteria included the followings: aged between 18 to 59 years old; closed and unilateral fracture; patients with displaced intra-articular calcaneal fracture (>2 mm) involving Sanders Type IIC and Type IIB; and patients have enough mental capacity to understand and answer questions in the evaluation scale. While the patients with medical contraindication or systemic infection or known local were excluded. Meanwhile, the open fractures, Sanders Type III, Type IV, and Type IIA fractures were also excluded.

### 2.3 Surgical procedure

Senior authors usually perform operation 5 to 12 days after injury (7.4 days on average). After admission, all patients had their limbs elevated and iced to avoid blisters and reduce swelling. All the patients underwent general anesthesia and then lay in the lateral position with affected foot.

In minimally invasive treatment group, the lateral calcaneal incision was made from the anterolateral corner of lateral malleolus tip through the tarsal sinus approach, passing through the tarsal sinus and calcaneocuboid joint, and finally extended to proximal cuboid bone. The anterior incision pointed to the lateral wall of calcaneus anterior process. After the reduction, Steinmann needle was inserted into the calcaneal process. After the reduction, the remaining large area defect was filled with allogeneic bone. After the Böhlers angle and Gissanes angle, the calcaneus length, width and height was fully restored under the C-arm fluoroscopy, then plate was implanted into front from posterior calcaneal incision through the percutaneous lateral calcaneal channel to the anterior calcaneal process. The anterior calcaneal process, subtalar articular surface and posterior part of calcaneus should be fixed with screws, and an extra compression screw pointed to the sustentaculum tali.

ORIF was adopted through utilizing the extended lateral approach with L-shape in conventional treatment group. A 10 to 12 cm incision was directly made in the bone to form the full-thickness flap. Once the reduction of calcaneal anatomic was completed, allogeneic bone would be applied for the reconstruction of calcaneal defect. Under C-arm fluoroscopy, calcaneal plates and screws (AO Synthes, Oberdorf, Switzerland) could also be utilized for the rigid fixation.

### 2.4 Postoperative protocol

The postoperative protocols of the 2 groups were similar. The calf isometric movements, limb elevation and active toe exercises were encouraged, and weight bearing was not allowed. The sutures could be closed after 2 weeks, and the problems of wound healing were recorded and then properly addressed. The passive and active range of motion trainings of the ankle joint, middle tarsal joint and the subtalar joint were began 2 weeks after the operation. The patient can only walk with their toes touching the ground with the help of walker for up to 6 weeks, and it is recommended that the patients kept their limbs elevated when sitting down and lying down.

### 2.5 Outcomes

In the process of outpatient follow-up, the radiographs were taken at 1, 3, 6, and 12 months. The x-ray investigation was implemented for the failure of internal fixation, the plate displacement, as well as the fracture healing. In the process of follow-up, these results were evaluated: the functional results, the satisfaction score of patient, the range of motion, as well as the related complications. The functional results involved the American Orthopaedic Foot and Ankle Score (AOFAS), Foot Function Index (FFI), and the score of pain. The postoperative complications involved infection, delayed union or nonunion, and stiffness, internal fixation loosening, as well as refracture.

AOFAS contains 4 systems of score, each of which includes 2 parts, one of which is subjective and it needs to be resolved through the patient; and other is objective and requires clinical examination. The most relevant score was given on the basis of the affected anatomical site. These 4 scoring systems involved the alignment examination for anatomical joint. Nevertheless, in the 4 systems, the function items are assigned into various subitems and different subitems. And total score is set to 100. The higher score represents better condition of patient. FFI was utilized for the measurement of the influence of foot pathology on pain, limited mobility and disability. FFI is a kind of self-management index, which involves 23 items and it is assigned into 3 subscales. The possible FFI score range from 0 to 100, with the lower the score the better the results. The pain intensity of patient is assessed with a 100-mm level visual analogue scale, 0 mm for no pain, 100 mm for extreme pain, and tgen compare between the groups.

### 2.6 Statistical analysis

The calculation of standard descriptive statistics was carried out, containing mean + standard deviation and the percentages. Mann–Whitney *U* test was applied for the analysis of baseline features between 2 groups, and the differences in all the dichotomous variables were determined with the Fishers exact Chi-Squared test. Between patient groups, the comparison of results was conducted with 2-tail Fisher test (for the categorical data) and unpaired Student t test (for the continuous data). The statistical significance could be defined as 5% (When *P* value is less than or equal to .05).

## 3 Discussion

For more than a century, the ideal selection of calcaneal fractures treatment has been controversial. Complications of minor wound and major wound is still a main problem because the skin on lateral wall of the calcaneus is vulnerable and thin, and the rates of marginal necrosis reported varies. Furthermore, recent evidence suggests that achieving consistency of the subtalar process does not appear to obviously affect the range of motion and clinical outcomes of the subtalar joint, which appears to be a specific objective after the anatomical reduction of the joint fragments. In theory, minimally-invasive surgery has the additional advantage of reducing the length of surgery and hospital stay. Hence, many authors believe that the minimally invasive internal fixation for calcaneal fractures should minimize the problems of soft tissue, with varying results. The minimally invasive surgery possesses an essential and growing function in treating the calcaneal fractures, but the related literature on this topic is limited. For our experiment, the main target was to compare the early prognosis of a group of the patients with Sanders type II fracture of calcaneus treated via minimally invasive surgery and ORIF.

## Author contributions

**Conceptualization:** Hongchang Yu.

**Data curation:** Dingshan Xue, Baozhen Lou, Rongrong Tan.

**Formal analysis:** Dingshan Xue, Baozhen Lou, Rongrong Tan.

**Funding acquisition:** Hongchang Yu.

**Investigation:** Dingshan Xue, Baozhen Lou, Rongrong Tan.

**Methodology:** Dingshan Xue.

**Project administration:** Hongchang Yu.

**Resources:** Hongchang Yu.

**Software:** Baozhen Lou.

**Supervision:** Hongchang Yu.

**Validation:** Baozhen Lou.

**Visualization:** Rongrong Tan.

**Writing – original draft:** Dingshan Xue.

**Writing – review & editing:** Hongchang Yu.
